# Protective Role of Native Rhizospheric Soil Microbiota Against the Exposure to Microcystins Introduced into Soil-Plant System via Contaminated Irrigation Water and Health Risk Assessment

**DOI:** 10.3390/toxins13020118

**Published:** 2021-02-05

**Authors:** El Mahdi Redouane, Majida Lahrouni, José Carlos Martins, Soukaina El Amrani Zerrifi, Loubna Benidire, Mountassir Douma, Faissal Aziz, Khalid Oufdou, Laila Mandi, Alexandre Campos, Vitor Vasconcelos, Brahim Oudra

**Affiliations:** 1 Water, Biodiversity and Climate change Laboratory, Department of Biology, Faculty of Sciences Semlalia, Cadi Ayyad University, Av. Prince My Abdellah, P.O. Box 2390, Marrakech 40000, Morocco; redouane.elmahdii@gmail.com (E.M.R.); soukainaelamranizerrifi@gmail.com (S.E.A.Z.); faissalaziz@gmail.com (F.A.); mandi@uca.ma (L.M.); oudra@uca.ac.ma (B.O.); 2 Bioactives, Health and Environement Laboratory, Biology, Environement & Health Research Unit, Department of Biology, Faculty of Sciences and technology, Moulay Ismail University, B.P. 509 Boutalamine, Errachidia 52000, Morocco; majidalahrouni@gmail.com; 3 CIIMAR, Interdisciplinary Centre of Marine and Environmental Research, Terminal de Cruzeiros do Porto de Leixões, Av. General Norton de MatosMatosinhos, 4450-208 Matosinhos, Portugal; jmartins@ciimar.up.pt (J.C.M.); acampos@ciimar.up.pt (A.C.); 4 Plant Biotechnology Laboratory BiotecV, Laayoune Higher School of Technology, Ibn Zohr University, 25 Mars P.B. 3007, Laayoune 70000, Morocco; l.benidire@uiz.ac.ma; 5 Laboratory of Chemistry, Modeling and Evironmental Sciences, Polydisciplinary Faculty of Khouribga (F.P.K), Sultan Moulay Slimane University, P.B. 145, Khouribga 25000, Morocco; douma_mountassir@yahoo.fr; 6 National Center for Studies and Research on Water and Energy (CNEREE), Cadi Ayyad University, B.P. 511, Av. Abdelkrim Elkhattabi, Marrakech 40000, Morocco; 7 Laboratory of Microbial Biotechnologies, Agrosciences and Environment (BioMAgE) Department of Biology, Faculty of Sciences Semlalia, Cadi Ayyad University, Av. Prince My Abdellah, P.O. Box 2390, Marrakech 40000, Morocco; oufdou@uca.ac.ma; 8 Departament of Biology, Faculty of Sciences, University of Porto, Rua do Campo Alegre, 4169-007 Porto, Portugal

**Keywords:** microcystins, *Vicia faba*, rhizospheric microbiota, *Rhizobium Leguminosarum*, plant growth, photosynthesis, nitrogen uptake, microcystins bioaccumulation, risk assessment

## Abstract

Microcystins (MCs) produced in eutrophic waters may decrease crop yield, enter food chains and threaten human and animal health. The main objective of this research was to highlight the role of rhizospheric soil microbiota to protect faba bean plants from MCs toxicity after chronic exposure. Faba bean seedlings were grown in pots containing agricultural soil, during 1 month under natural environmental conditions of Marrakech city in Morocco (March–April 2018) and exposed to cyanobacterial extracts containing up to 2.5 mg·L^−1^ of total MCs. Three independent exposure experiments were performed (a) agricultural soil was maintained intact “exposure experiment 1”; (b) agricultural soil was sterilized “exposure experiment 2”; (c) agricultural soil was sterilized and inoculated with the rhizobia strain *Rhizobium leguminosarum* RhOF34 “exposure experiment 3”. Overall, data showed evidence of an increased sensitivity of faba bean plants, grown in sterilized soil, to MCs in comparison to those grown in intact and inoculated soils. The study revealed the growth inhibition of plant shoots in both exposure experiments 2 and 3 when treated with 2.5 mg·L^−1^ of MCs. The results also showed that the estimated daily intake (EDI) of MCs, in sterilized soil, exceeded 2.18 and 1.16 times the reference concentrations (0.04 and 0.45 µg of microcysin-leucine arginine (MC-LR). Kg^−1^ DW) established for humans and cattle respectively, which raises concerns about human food chain contamination.

## 1. Introduction

Over the last decades, bloom-forming cyanobacteria have been increasing in frequency and intensity in eutrophic freshwater ecosystems worldwide, in which they produce and release, various deleterious secondary metabolites (i.e., cyanotoxins), into the extracellular environment [1,2,3]. Microcystins (MCs) are the most widespread and prevalent cyanotoxins that have potent toxicity and more than 250 congenrs described [4,5,6,7]. MCs are hepatotoxic cyclic heptapeptides produced by several bloom-forming cyanobacteria belonging to several genera such as *Microcystis*, *Nostoc*, *Oscillatoria*, *Planktothrix* and *Dolichospermum* [8,9,10,11,12,13]. Owing to their ring structure, MCs are chemically stable under several field and laboratory conditions with half-life ranging from 0.4 to 22 days [14,15,16,17,18,19] and total concentrations varying from less than 1 µg·L^−1^ to 29,000 µg·L^−1^ in surface waters [20,21,22,23,24,25,26,27,28,29,30]. Dissolved MCs in water bodies are recognized as a major public health hazard and disrupting agents of certain fundamental ecological processes [31,32,33]. Likewise, MCs occurrence in irrigation ponds and reservoirs, may affect negatively the yield, productivity and nutritional quality of agricultural crops that rely mainly on this water resource [34,35,36,37,38]. Moreover, MCs induce acute oxidative stress and several toxic effects on plant biochemical and physiological functions, including: (a) plant growth and development; (b) photosynthesis; (c) carbon dioxide fixation; (d) sucrose biosynthesis; (e) starch storage; (f) nitrogen uptake; (g) hormone transport and translocation; (i) mitosis and DNA functions [3,39,40]. MCs introduced into soil via irrigation, seem to be bioavailable to soil-crop systems during a long-term period on account of their adsorption onto clay-humic particles, with half-life ranging from 1 to 17.8 days. Furthermore, MCs persistence is depending on soil chemical composition and microbial activity [14,39,41,42,43,44,45], with total concentrations varying from 1.6 µg Kg^−1^ to 187 µg Kg^−1^ in soil compartment [35,46,47,48]. Indeed, in certain regions where water resources are scarce, MC-containing waters are largely used to irrigate crops, which leads to food chain contamination and thus results in human exposure to cyanotoxins; since several studies have reported the bioaccumulation of MCs in plant tissues, some of which focused on edible plants [35,36,38,47,49,50,51,52,53]. However, in order to overcome these issues, plants need to develop protection mechanisms against cyanotoxins stress. In general, the ability of crop plants to cope with biotic stress is, in a big part, related to plant-growth-promoting rhizobacteria (PGPR) as it was already demonstrated in our previous works: (a) MC-tolerant rhizobia strains protect faba bean plants and improves nitrogen assimilation when irrigated with MC-containing water; (b) several bacterial communities of *Medicago sativa* rhizosphere (*Clostridia*, *Opitutae* and bacteria related with *Betaproteobacteria*) were stimulated in response to MCs exposure [54,55,56]. That is to say, MCs can be removed from the rhizosphere compartment according to several processes, among which microbial degradation remains a major removal process to alleviate contamination by these hepatotoxins in agroecosystems [31,41,42,45]. Questions arise about the benefic role of rhizospheric microbiota to protect plants from the harmful effects of cyanotoxins introduced via irrigation water and to enhance the productivity and quality of crop plants under MCs exposure. Therefore, the aim of the present study was to investigate, for the first time, whether plants grown in the presence of natural rhizospheric microbiota can cope with MCs stress as compared to those grown in sterilized soil. In particular the following parameters were evaluated: (a) plant growth and photosynthesis; (b) nitrogen uptake; (c) toxin uptake and accumulation; and (d) impact on human and animal health. Moreover, this paper suggested the use of symbiotically MC-tolerant and degrading bacteria as a new bioremediation tool and environmentally-relevant procedure to mitigate MCs phytotoxic effects and transfer to the crop plants and thus to the human and animal food chain.

## 2. Results

### 2.1. Identification and Quantification of MCs in Microcystis Biomass

**Figure 1:**
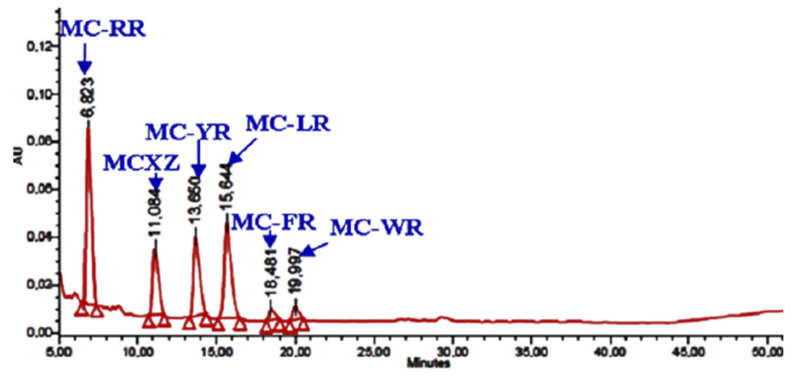
High-performance liquid chromatography-photodiode array detection (HPLC–PAD) chromatogram of freeze-dried material of *Microcystis aeruginosa* bloom collected from Lalla Takerkoust lake-reservoir in 2008, Marrakech, Morocco.

The total MCs content was evidenced by high-performance liquid chromatography (HPLC) analysis of *Microcystis* biomass as 1160 µg·g^−1^ equivalent microcystin-leucine arginine (MC-LR) in dry weight. The chromatogram of *Microcystis* crude extract exhibited six different variants of heptapeptide MCs with different L-amino acids in positions 2 (X) and 4 (Z) of the cyclic peptide (Figure 1). MC-LR (leucine arginine) (24.49%), -RR (arginine arginine) (35.65%), -YR (tyrosine arginine) (18.62%), -XZ (unknown variant) (13.02%), -WR (tryptophane arginine) (4.96%) and -FR (phenylalanine arginine) (3.26%).

### 2.2. Effects of MCs Extract on Faba Bean Plants Growth

Upon conducting a visual inspection of *V*. *faba* plants exposed for 28 days to cyanobacterial bloom extract containing 2.5 mg·L^−1^ MC-LR, no deleterious effects in faba bean plants (e.g., morphological changes, necrosis or chlorosis) were perceptible compared to the controls. At the end of the experiment, the effects of MCs on faba bean growth were studied by comparing the means of stem length (SL), shoot dry weight (SDW) and root dry weight (RDW) between the control and treatment groups at different exposure experiments (Table 1). The obtained results showed that no significant differences (*p* < 0.05) were observed between control and treatment groups for plants grown in intact soil. In addition, in this soil, the reduction rate was 0% for all growth parameters studied (Table 1). The growth of plants cultivated in sterilized soil was more affected by MCs than those cultivated in inoculated soil. Indeed, exposure to water containing 2.5 mg·L^−1^ MCs for 28 days decreased significantly (*p* < 0.05) the SL of faba bean plants grown in sterilized soil; the reduction rate was up to 30.96%. However, the SL of plants cultivated in inoculated soil was slightly reduced by about 9.6% (Table 1). Likewise, the SDW reduction was 0% for plants grown in intact soil, whereas in plants cultivated in sterilized and inoculated soils, the reduction rates were 7.75 and 13.46% respectively. Regarding RDW, despite that roots are in direct contact with MCs in soil, root mass was less affected by MCs than shoot mass. Overall, no significant differences in RDW were observed between control and treatment groups for all exposure experiments. Moreover, the reduction rate was 0% for plants in intact and inoculated soils and 5.55% in sterilized soil (Table 1). These results suggest that the agricultural soil microbiota and rhizobia strain RhOF34 had a protective role on faba bean plants exposed to the phytotoxic effect of MCs.

### 2.3. Effects of MCs extract on Faba Bean Photosynthetic Capacity

**Figure 2:**
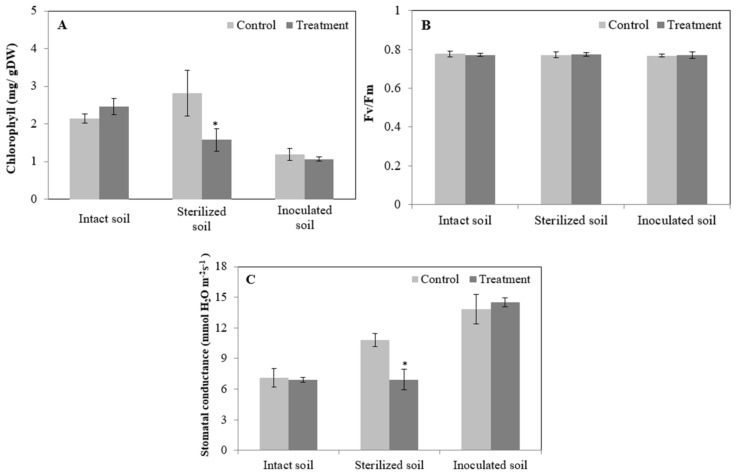
Photosynthetic capacity was assayed as chlorophyll (a + b) content (**A**), chlorophyll fluorescence Fv/Fm (**B**) and stomatal conductance (**C**) of *Vicia faba* plants exposed to MCs crude extract containing 2.5 mg·L^−1^ of MCs for 28 days and submitted to three independent exposure experiments. Significant differences (*p* ≤ 0.05) between control and treatment groups are indicated by (*).

The effect of MCs on photosynthetic capacity was evaluated as chlorophyll fluorescence, chlorophyll content (a + b) and stomatal conductance. Compared to the control, no significant changes were registered in photosynthetic capacity in relation to the intact and inoculated soils (Figure 2A–C). Regarding sterilized soil, the exposure of faba bean plants to contaminated water significantly decreased chlorophyll (a + b) content and stomatal conductance but did not affect chlorophyll fluorescence (Figure 2A–C). Overall, Fv/Fm was not affected by the contaminated water in any exposure experiment; its value remained the same (≈0.78) for control and treatment groups (Figure 2B).

### 2.4. Effects of MCs Extract on Nodulation and Nitrogen Uptake of Faba Bean Plants

**Figure 3:**
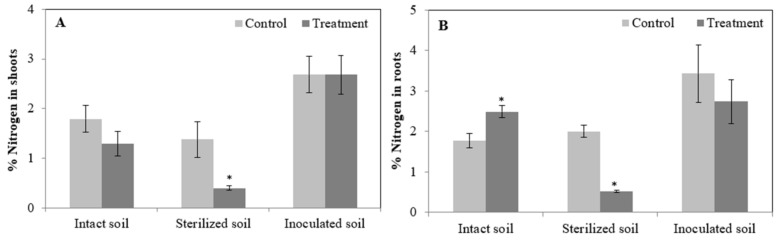
Nitrogen content in shoots (**A**) and roots (**B**) of *Vicia faba* plants exposed to MCs crude extract (2.5 mg·L^−1^ MCs) for 28 days and submitted to three independent exposure experiments. Significant differences (*p* ≤ 0.05) between control and treatment groups are indicated by (*).

In order to demonstrate the effect of MCs on nodulation capacity and the effectiveness of nitrogen fixation in nodules, TNN was counted and nitrogen content was determined in shoots and roots of faba bean plants grown in different soils. The obtained results showed that the exposure to MCs contaminated water (2.5 mg·L^−1^ MCs) resulted in significant changes of nitrogen content of faba bean shoots and roots (Figure 3A,B). Overall, nitrogen uptake of N-fertilized plants (sterilized soil) was more affected by MCs than those relying on symbiotically fixed nitrogen (intact and inoculated soils). Indeed, the exposure to contaminated water containing 2.5 mg·L^−1^ MCs significantly decreased nitrogen content in shoots and roots of *Vicia faba* plants grown in sterilized soil. However, no significant decrease was observed in plants grown in the presence of rhizobial strains (intact and inoculated soils) (Figure 3A,B). In addition, compared to the control, a significant increase in TNN and nitrogen content was observed in roots exposed to MCs in relation to the intact soil (Table 1 and Figure 3B). Whereas no significant changes in TNN and nitrogen content were registered for faba bean plants inoculated with the rhizobia strain RhOF34. These results indicate that nodules elicited by this strain are efficient to alleviate the adverse effects of MCs on faba bean growth parameters (Table 1 and Figure 3A,B).

### 2.5. Accumulation of Microcystins in Vicia faba Shoots and Roots

Table 2 shows the enzyme-linked immunosorbent assay (ELISA) results regarding the amount of MCs accumulated in faba bean tissues (stem/leaves and roots); this bioaccumulation is assessed in shoots via bioconcentration factor (BCF), estimated daily intake (EDI) and factor exceeding TDI which is a comparison between the EDI of MCs and the recommended consumption doses limited in 0.04 and 0.45 µg·kg^−1^ BW Day^−1^ for humans and farm animals (cattle) respectively. In intact and sterilized soils, faba bean plants were exposed to 2.5 mg·L^−1^ MCs for 28 days; the accumulation of MCs in faba bean shoots was about 8 and 13.1 μg·Kg^−1^ DW with a capacity to bioconcentrate MCs by at least 3.2- (moderate) and 5.2-fold (high), respectively. On the other hand, the EDI of MCs for plants grown in intact ans sterilized soils, was about 0.053 and 0.087 μg·Kg^−1^ DW for humans; and 0.32 and 0.52 μg·Kg^−1^ DW for cattle respectively, which was 2.18- and 1.16-fold higher than the recommended tolerable daily intake, for plants from sterilized soil, respectively. This would pose a serious health hazard by consuming MC-contaminated faba beans or eventually via dairy and beef products issued from MC-infected animals feeding on MC-contaminated silage. The health risk is much higher when faba bean plants were grown in sterilized soil as compared to intact soil which suggests that native rhizospheric microflora may play a crucial role in keeping MCs content under the recommended limits of daily consumption of plants used as silage and animal feed. 

## 3. Discussion

In our experiments, after 28 days of faba bean exposure to cyanobacterial bloom extract containing 2.5 mg·L^−1^ MCs, no visual morphological changes were observed and no significant differences were registered in root dry weight for all exposure experiments. This is in discordance with what has been reported from experiments with low toxin concentrations. For instance, Zhu et al. (2018) [36] reported that after 7 days’ exposure to 100 and 1000 μg·L^−1^ MCs, visible changes in *Cucumis sativus* L. were observed such as necrosis and chlorosis in the leaves, besides a sharp decline on the size of root and shoot organs and only 10 μg·L^−1^ MCs reduced root dry weigh by about 29%. Moreover, Llana-Ruiz-Cabello et al. (2019) [57] reported a decrease in fresh weight, morphological changes and deleterious effects on leaves (i.e., necrosis) of spinach plants exposed to 50 μg·L^−1^ pure MC-LR. However, contrarily, in line with the present study, Bettencourt-Oliveira et al. (2016) [58] reported the absence of morphological changes between the treatment and control groups in lettuce plants (i.e., root, shoot) exposed to 0.65, 2.5, 6.2 and 13 μg·L^−1^ total MCs. Likewise, in our previous study, we reported no visual morphological changes perceptible in faba bean plants exposed to 50 and 100 μg·L^−1^ total MCs for 28 days [54].

Regarding shoot growth, SL and SDW were not affected by MCs exposure (reduction rate 0%) when faba bean plants were grown in intact soil. In contrast, noticeable decreased growth was observed for faba bean plants grown in sterilized and inoculated soils. MCs reduced shoot growth in sterilized soil followed by inoculated soil with reduction rates up to 30.96 and 9.6% for SL; and 7.75 and 13.46% for SDW respectively. This finding is in total agreement with our previous work where inoculation with rhizobial strains enhanced plant growth under MCs exposure (100 μg·L^−1^ MCs for 28 days) [55]. Indeed, the SDW reduction was 27 % on average for plants fed with nitrogen, whereas in inoculated plants, the reduction rate was 15 % (not significant at *p* < 0.05) [55]. The reduction of shoot growth under MCs exposure was also reported by Zhu et al. (2018) [36] where the authors remarked a decrease in SDW of cucumber plants after exposure to 100 and 1000 μg·L^−1^ MCs for 7 days. Furthermore, interestingly, in another study, it was reported that lettuce plants, treated with 5 and 10 μg·L^−1^ MCs were shorter, had fewer leaves per head and weighed less than the control group. Likewise, it was reported that carrot plants, exposed to 1, 5 and 10 μg·L^−1^ MC showed a decrease in total mass and root diameter as compared to the control group [35].

In our work, chlorophyll fluorescence of faba bean plants was not affected by 2.5 mg·L^−1^ MCs in any of the exposure experiments and all values reported were above 0.76. Indeed, the chlorophyll content and stomatal conductance significantly decreased under MCs exposure when faba bean plants were grown in sterilized soil, this decrease did not significantly affect photosynthesis performance in which Fv/Fm ratio was around 0.78 for control and treatment groups. Regarding plants grown in intact and inoculated soils, exposure to MCs had no negative effects on any of the photosynthetic studied parameters (chlorophyll content, stomatal conductance and chlorophyll fluorescence) (Figure 2A–C). In agreement with the present results, Llana-Ruiz-Cabello et al. (2019) [57] reported that chlorophyll fluorescence of spinach and lettuce plants were not affected by exposure to 10 and 50 μg·L^−1^ of pure MC-LR for 21 days in which Fv/Fm values were between 0.75 and 0.85. Moreover, Machado et al. (2017) [59] and Bittencourt-Oliveira et al. (2016) [58] reported an increase in the net photosynthetic rate in carrot and lettuce plants exposed to cyanobacterial crude extract containing from 0.65 to 50 μg·L^−1^ of total MCs. This increase in the photosynthetic capacity under MCs exposure was inspected by the authors, in one hand, as a mechanism of lettuce plants to produce additional substrates to supply chemical energy and biosynthesize key enzymes and antioxidant molecules involved in the protection from oxidative stress induced by MCs [58]. On the other hand, Machado et al. (2017) [59] reported an increase in all minerals recognized as essential to photosynthesis performance (K, Mg, Fe, Mn, Cu and Zn) in carrot plants exposed to MCs crude extract containing 10 and 50 μg MC-LR L^−1^, hence the observed increase in Fv/Fm ratio, since they participate in several functions involved in the photosynthesis process. Moreover, the Fv/Fm ratio reflects an optimal potential quantum efficiency of PSII with values of around 0.8 measured for majority of the plants, whereas values significantly lower than this could indicate that the plant is under stress conditions [60]. Therefore, we can state that MCs at 2.5 mg·L^−1^ do not impair photosynthesis performance in faba bean plants in all exposure experiments (Figure 2B) which agrees with the absence of significant decrease in SDW and RDW (Table 1). The results found in the present work are in disagreement with our previous study where MCs at concentrations of 50 and 100 µg·L^−1^ MC-LR significantly decreased faba bean SDW, RDW and chlorophyll fluorescence [54]. 

It is well known that the different responses of plants to MCs may be related to plant species, plant genetic, plant growth stage, toxin variant, toxin nature (purified or crude extract), exposure dose and time, microbial and biochemical composition of agricultural soil in which the plant subject is studied [43,45,55,57,61,62,63,64,65]. Interestingly, it is important to note that: (1) plant cultivars used in our studies were Aguadulce [54] and Alfia 5 (the present study); (2) cyanotoxins crude extracts, used in irrigation water, were prepared by the dilution of a toxic *Microcystis* bloom extract collected from Lalla Takerkoust lake-reservoir in 2005 [54] and in 2010 (the present study). It is also known that other potential cyanobacterial bioactive metabolites could be present in the crude extract along with the main toxin MC-LR. Thus, the biological activity of the crude extract may not be confined to MCs but also to the interaction of other non-identified cyanobacterial bioactive metabolites with the plant. Since only MCs were quantified in the crude extract as the main bioactive compounds, the results are referred only to these toxins. Therefore, according to this work, we can state that the dissimilarities observed in the response of faba bean plants to cyanotoxins crude extract exposure ([54] and the present study) are due to faba bean variety, cyanotoxins crude extract composition and microbial and biochemical composition of soil in which the plant was grown. In this study, the exposure to MCs led to the failure of faba bean plants, grown in sterilized soil, to uptake nitrogen, most likely due to the absence of soil natural microbiota and/or rhizobial strains as recently confirmed by our previous work; where we demonstrated that nitrogen content of N-fertilized plants was more affected by MCs than those relying on symbiotically fixed nitrogen and that nodulation and nitrogen content were significantly impaired by MCs, with the exception of plants inoculated with the most tolerant rhizobia strain RhOF125 [55]. In the light of this work, it can be hypothesized that the increase in nitrogen content could indicate an improved uptake capacity of plants grown in agricultural soil containing natural rhizospheric microbiota and/or rhizobia strains, hence the possible raise in growth parameters and physiological performance (e.g., photosynthesis).

On the other hand, MCs must be considered when evaluating the health risk in edible plants for both humans and farm animals. Therefore, we described the accumulation of MCs in faba bean roots and shoots by calculating the BCF and EDI (Table 2). In this light, MCs were accumulated 3- and 2-fold higher in roots than in shoots, in both plants from intact and sterilized soils, respectively. Several crops have been found to accumulate a considerable amount of MC in roots than in shoots according to many studies [47,49,66,67]. It is also reported that plants uptake MCs dissolved in extracellular environment and accumulate them with a BFC 3-fold higher than MCs dose in irrigation water [38,47,66]; likewise, faba bean shoot seems to show a high BFC, 5-fold higher in sterilized soil and only 3-fold higher in intact soil which suggests that in absence of soil microflora, MCs escape from biodegradation process and become more available to plants, hence their accumulation in stem/leaves organs at a rate exceeding the tolerable daily intake (TDI) established for humans and farm animals in 0.04 [4] and 0.45 µg kg^−1^ BW. Day^−1^ [68] respectively. Several studies have reported the accumulation of MCs in edible crops such as lettuce, cucumber and carrot, at a concentration rate exceeding the safety threshold set for humans by WHO [36,51,53]. As for livestock, Crush et al. (2008) [69] have reported that clover plants, grazed by cows, accumulate 21 mg MCs Kg^−1^ DW which exceeds the TDI set for cattle; hence, the potential contamination of the human food chain (milk, meat). Nevertheless, studies about transfer of MCs to animal products like milk and meat are scarce. Orr et al. (2001, 2003) [70,71] have conducted a similar study by supplying the cells of a MC-producing cyanobacterium, *Microcystis aeruginosa*, in drinking water to dairy and beef cattle and no problematic concentrations of MCs were detected in milk and beef liver. Despite these findings, studies on EDI of MCs for farm animals and eventual transfer of these toxins to dairy and meat products are to further investigate; besides the role of native microflora in mitigating plant uptake and accumulation of MCs through biodegradation process.

Indeed, it was previously reported that a *Rhizobium* sp. may participate in the degradation of MC-LR [72]; similarly, another author reported that a strain of *Rhizobium selenitireducens* could degrade MCs and the gene involved in the biodegradation process was fully identified [73]. Moreover, El Khalloufi et al. 2016 [56] demonstrated that the growth of some bacterial species of rhizospheric microbiota associated to *Medicago sativa* crop such as *Clostridia*, *Opitutae* and other bacteria related to *Betaproteobacteria*, were stimulated under MCs exposure. To our knowledge, once MCs are introduced into agricultural soils via irrigation water, their bioavailability to plants depends on toxin variant, soil nature, soil chemical composition and soil rhizospheric microbiota. Therefore, the sorption of MCs onto soil particles and its degradation by native flora reduce its uptake by the plants [41,44,45,49,50,62,66,69,74]. Furthermore, it was reported that: (1) plants cultivated without soil in hydroponic systems are more exposed to free MCs whose accumulation in plant organs was extremely higher compared to soil-grown plants [40,47] and (2) plants cultivated in soils with higher microbial activity results in the rapid degradation of MCs [41,45]. These two examples corroborate the present finding where faba bean plants grown in sterilized soil accumulated more MC than those grown in intact soil (Table 2).

## 4. Conclusions

MCs, at a concentration of 2.5 mg·L^−1^, have adverse phytotoxic effects on faba bean plants cultivated in sterilized soil, in which a significant decrease was observed on growth parameters in terms of SL and SDW and photosynthesis performance in terms of chlorophyll content (a + b) and stomatal conductance. On the other hand, no significant changes in these parameters were noted for faba bean plants cultivated in both intact and inoculated soils, in the presence of native rhizospheric microbiota and *Rhizobium Leguminosarum* RhOF34 respectively; likewise, MCs have significantly decreased nitrogen uptake in sterilized soil (microorganism-free soil) compared to intact and inoculated soils. Moreover, MCs are less accumulated and bioconcentrated in root and shoots organs in intact soil compared to sterilized soil, suggesting that microbial activity in the rhizosphere, including rhizobial symbiosis, is pivotal for removing MCs and alleviating its uptake and accumulation in plant tissues to prevent its entering to the agricultural food supply which raises sanitary concerns over its safety for consumption by humans and livestock. In this light, using symbiotically MC-tolerant and degrading microorganisms or developing new strategies to enhance and boost microbial degradation of MCs in the soil-plant system constitutes a friendly ecological process to manage and mitigate the hazardous impact of MCs introduced into agricultural soils via irrigation water contaminated with MC-producing cyanobacteria blooms.

## 5. Materials and Methods

### 5.1. Bloom Sampling

*Microcystis* bloom was collected with a 27-µm mesh phytoplankton net from Lalla Takerkoust lake-reservoir, Marrakech, Morocco (31°36′ N, 8°2′ W) in august 2005. A *Microcystis aeruginosa* strain was identified as the dominant species by light microscopy according to diverse morphological criteria [75,76]. Bloom sample was freeze-dried and stored at −20 °C for MCs extraction and HPLC analysis.

### 5.2. Extraction, Identification And Quantification of MCs from Bloom Material

MCs were extracted, as described by Douma et al. (2010) [77], from 2 mg of lyophilized cyanobacteria biomass in 1 mL of 70% aqueous methanol (*v/v*). Three successive extractions were carried out to assure total MC recovery and obtained extracts were pooled together and vacuum dried by rotary evaporation at 45 °C. To analyze MCs, the dried extracts were first suspended in 70% methanol (*v/v*) and filtered through a GF/C filter. MCs analysis was performed by high-performance liquid chromatography-photodiode detection array (HPLC-PAD), with an equipment (Waters, model 2695) provided with a photodiode array detector (model 996) and fitted with a Chromolith C18 column (250 mm × 4.6 mm, 5 mm.). The mobile phase was a discontinuous gradient of water and acetonitrile, both with 0.05% trifluoroacetic acid (*v/v*). The analysis was run at a flow rate of about 1 mL min^−1^ and MCs were identified according to their UV-spectra and retention time. Quantification of each MC variant was carried out using the appropriate standards: MC-RR, -YR and -LR (Calbiochem, Darmstadt, Germany). All chemicals were of chromatographic grade (ScharlauChemie Barcelona, Spain); MC-WR and -FR were purified in the Autonomous University of Madrid laboratory.

### 5.3. Soil Sampling and Preparation 

**Figure 4:**
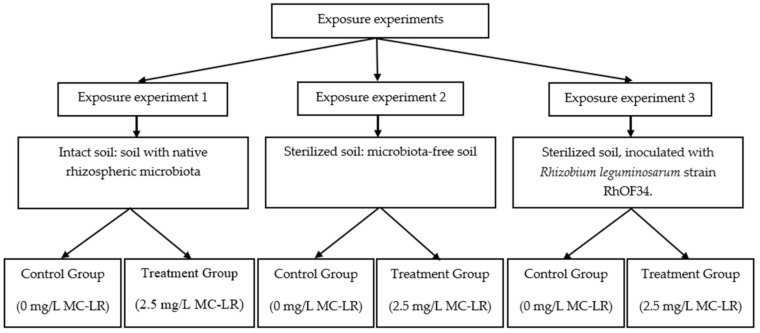
Experimental design of greenhouse trial; three independent exposure experiments were carried out: intact soil (Exposure experiment 1) and sterilized soils; without a source of microorganism inoculum (Exposure experiment 2) and inoculated with Rhizobium leguminosarum strain RhOF34 (Exposure experiment 3). 5 pots per group of treatment or control; 4 plants per pot.

Soil samples were collected randomly at 20 cm depth from mono-cropped faba bean (*Vicia faba* L., 1753) culture over the growing season in February, 2018. The sampling field was located in Demnate city (31°43′52″ N, 7°02′10″ W); the soil was pesticide-free and had no known history of any chemical application that might disrupt the structural and functional development of native microflora. After being brought back to the laboratory, plant debris and pebbles were removed, soil clods were crumbled and mixed into a uniform homogeneous soil. Hereafter, the soil was divided into three equal parts; one third remained intact; this group contained native microflora associated to faba been rhizosphere, the two remaining thirds were sterilized in furnace muffle at 150 °C for 3 h and subsequently splitted into two equal parts; one part was kept microorganism-free and the other one was inoculated by a rhizobia strain, previously identified as *Rhizobium leguminosarum* RhOF34 [78]. Then, homogenized soils were transferred into 10 pots of 5 L volume for each of the three exposure experiments (Figure 4); 5 replicates *per* treatment and control group. For simplification, throughout this work the intact agricultural soil containing natural rhizospheric microbiota will be referred to as “intact soil,” sterilized agricultural soil will be referred to as “sterilized soil” and sterilized agricultural soil inoculated by rhizobia strain RhOF34 will be referred to as “inoculated soil” (Figure 4).

### 5.4. MCs Aqueous Crude Extract Preparation 

MCs crude extract was prepared as described by Lahrouni et al. (2012) [79]. Briefly, bloom material was thawed and subjected to ultrasounds at 3 KHz during 5 min in order to release total intracellular MCs trapped in *M*. *aeruginosa* cells. Afterwards, the extract was centrifuged at 4000× g for 12 min to remove cellular debris. Finally, the supernatant containing total MCs was recovered in order to prepare the irrigation solution at the concentration of 2.5 mg MC L^−1^. 

### 5.5. Plant Culture and Exposure Experiments 

Commercial seeds of a cultivar of faba bean (*Vicia faba* L. var. alfia 5) were purchased for the greenhouse trial. Homogeneous seeds of same size, shape and color were surface-sterilized as described in our previously reported methods [55]. Briefly, seeds were shaken vigorously in sodium hypochlorite (6°) for 15 min, washed thoroughly with sterile distilled water and soaked for 8 h. Water-imbibed seeds were placed in sterile petri dishes on a filter paper and incubated for germination at 25 °C during 48 h. Pre-germinated seedlings with 1 cm of root length were picked up for exposure experiments. The greenhouse trial was conducted over the period March–April, 2018. Four seedlings were sown 4 cm deep at 5 cm apart in each pot. Each seedling was inoculated with 2 mL of a suspension of the rhizobia strain RhOF34 identified as *Rhizobium leguminosarum* [78] and containing around 109 CFU/mL of liquid inoculant in the soil of exposure experiment 3, whereas the two other groups served for exposure experiments 1 (intact soil) and 2 (sterilized soil) remained intact. After expansion of first leaves, seedlings were exposed to MCs crude extract at 2.5 µg·mL^−1^ added to distilled water. Plants were irrigated daily to field capacity with 50 mL of distilled water, supplemented by MCs at 2-days intervals; control groups were irrigated with MC-free water for each of the exposure experiments. A nitrogen-free solution [80] containing 10 mM NH_4_NO_3_ as a nitrogen source was added for the plants of exposure experiment 2 every 3 days. Plants were grown under MCs chronic exposure for 4 weeks under natural conditions of light, humidity, temperature and photoperiod in Marrakech city.

### 5.6. Plant Harvest and Growth Parameters

After 28 days exposure to cyanobacterial crude extract containing MCs, faba bean plants were pulled out gently of the soil and roots were washed with distilled water to remove soil particles. Only after this cleaning step, half of the plants (10 plants) were taken to measure growth parameters: shoots length (SL) and total nodules number (TNN). After desiccation at 70 °C during 72 h, the dry weight of shoots (DWS) and roots (DWR) were determined. An average of growth parameters was estimated from a total of 10 plants per experimental group. For the other plants, shoots and roots were separated and subsequently submerged in liquid nitrogen, stored at −20 °C and then freeze-dried for further analysis of chlorophyll content and MCs bioaccumulation.

### 5.7. Photosynthesis Parameters 

On the 28th day of exposure experiments, before plant harvest, chlorophyll fluorescence and stomatal conductance of fully expanded leaves were measured under well-watered conditions. After plant harvest, chlorophyll (a + b) content was determined using freeze-dried leaves in order to evaluate photosynthetic activity which provides energy for plant growth and development.

#### 5.7.1. Chlorophyll Fluorescence 

Chlorophyll fluorescence was assayed by flashing dark-adapted leaves (10 leaves per treatment) at a light intensity of 3500 μmole·photons·m^−2^ s^−1^ using a portable fluorometer (Handy Plant Efficiency Analyser, Hansatech Instruments Ltd) as described by Lahrouni et al. (2013) [54]; leaves were dark-adapted for about 20 min prior to measurement. Photosystem-II (PSII) effective quantum yield was estimated by the ratio (Fm − F_0_)/Fm, also known as Fv/Fm, in which F_0_ corresponds to zero level fluorescence when PSII centers are open and Fm corresponds to maximum fluorescence when PSII centers are closed. The fluorescence values were calculated automatically, using Handy PEA v 1.3 software.

#### 5.7.2. Stomatal Conductance

Stomatal conductance (gs) was assayed on dorsal side of leaves (10 leaves per treatment) during daytime between 11:00 and 14:00 h, using a portable porometer (Leaf Porometer, Decagon Devices, Pullman, WA, USA) as described by Zeppel et al. (2012) [81]. Leaves were allowed to equilibrate within the cuvette for at least 30 s, until the gs value remained stable at a temperature of 25 ± 1 °C. Prior to measurement; the leaf porometer was equilibrated to room temperature for 2 h.

#### 5.7.3. Chlorophyll Content 

Chlorophyll (a + b) content in leaves was analyzed in order to assess the impact of MCs exposure on photosynthetic apparatus. Chlorophylls a and b were extracted in the dark and dosed according to the methodology described by Geider and Osborne (1992) [82]. Briefly, 25 mg of lyophilized leaves were crushed and homogenized in 3 mL of aqueous acetone 90% (*v/v*). The homogenate was centrifuged at 10,000× g for 5 min and the pellet was extracted twice with 0.5 mL of aqueous acetone 90% (*v/v*). Following this step, supernatants were pooled together to a final volume of 4 mL and served for dosing total chlorophyll (a + b) per gram of plant material dry weight (DW). The concentration of chlorophylls a and b was calculated from the absorbance at 664 and 647 nm using the equations given below [83]:

Total chlorophyll = chlorophyll a + chlorophyll b, with:(1)

Chlorophyll a (mg g^−1^ DW) = [(12.7 × A664) − (2.69 × A647)] µg·mL^−1^ × [(4 mL × mg)/(0.025 g DW × 103 µg)] (2)

Chlorophyll b (mg g^−1^ DW) = [(22.9 × A664) − (4.68 × A647)] µg·mL^−1^ × [(4 mL × mg)/(0.025 g DW × 103 µg)] (3)

### 5.8. Nitrogen Content 

Nitrogen content in roots and shoots was determined by Kjeldahl procedure as described by Lahrouni et al. (2012) [79]. Briefly, 0.5 g of plant material dry weight was digested with 10 mL of sulfuric acid (98%) and 1 g of a catalyst mixture (K_2_SO_4_, CuSO_4_. 5H_2_O and Se) as a boiling point elevator, at 420 °C for 90 min. After complete mineralization, the digest solution was brought to 100 mL with distilled water; 40 mL of this solution was transferred to Kjeldahl bottles containing few drops of concentrated NaOH (8 N) and the resulting distillate was collected on boric acid and titrated with 0.05 N sulfuric acid.

### 5.9. Extraction and Quantification of Total Mcs from Plant Material

Total MCs in plant tissues, shoots and roots, were quantified using enzyme-linked immunosorbent assay (Microcystins/Nodularins (ADDA) kit, 520011, Eurofins Abraxis, Warminster, PA, USA) as a cost-effective and widely-used tool to determine total MCs content in different kind of matrixes at low concentrations. Plant extracts of stem/leaves and roots tissues from the exposure experiments were prepared prior to ELISA analysis as described by Saqrane et al. (2009) [50] with slight modifications. The lyophilized faba bean tissues (stem, leaves and roots) were ground with a mortar to fine powders and aliquots of 100 mg of roots and 250 mg of stem/leaves were homogenized with 5 mL and 10 mL of aqueous methanol 70% (*v/v*) respectively. The slurry was sonicated for 5 min and then centrifuged at 10,000× g at 4 °C for 15 min. The pellets were re-extracted twice as before and then all supernatants were pooled together and semi-purified on C18 solid phase extraction cartridges (LiChrolut® RP-18, 40–63 µm, 1000 mg 6 mL, Sigma-Aldrich, Munich, Germany). MCs were eluted with 3 mL methanol and the eluate was vacuum dried with a rotary evaporator at 45 °C and resuspended into 1 mL of ultrapure water. The ELISA test was carried out according to the user’s guide provided by the company and absorbance was read at 450 nm using a Multiskan FC microplate photometer from TermoFischer Scientific (Srasbourg, France).

### 5.10. Risk Assessment

#### 5.10.1. Bioconcentration Factor of MCs (BCF)

A bioconcentration factor (BCF) between 0 and 3 refers to a “Low” microcystins bioconcentration; between 3 and 3.7 indicates a “Moderate” microcystins bioconcentration and stipulates a “High” microcystins bioconcentration when BCF is > 3.7. When the BCF is less than 0, it means that MCs concentration was likely diluted because biomass gain was greater than microcystin bioconcentration, although we cannot exclude the possibility that microcystins were depurated within the plant tissues [58]. The BCF of MCs in faba bean was calculated using the equation given below according to the European Union and US Environmental Protection Agency [84]:

BCF = MC_tp_/MC_tw_, where MC_tp_ is the MCs detected in faba bean (μg·kg^−1^), while MC_tw_ is the MCs in irrigation water (μg·L^−1^). (4)

#### 5.10.2. Estimated Daily Intake (EDI) 

The estimated daily intake (EDI) of MCs per 40 g of faba beans consumed by an average-sized adult (60 kg) was calculated from the amount of the toxin in the vegetables during bioaccumulation process using the equation below [51] and compared to tolerable daily intake set by the World Health Organization (WHO) at 0.04 µg per kilogram of body weight per day [4]. The amount of MC in stem/leaves organs was used to calculate EDI of MCs in faba bean seeds as a matter of supposition.

EDI _faba bean_ = MC_40 g_^−1^/BM_60 kg_, Where MC_40 g_^−1^ represents the amount of MC accumulated per 40 g of faba bean and BM_60 kg_ is the body mass of an adult. (5)

On the other hand, faba bean shoots are widely used as silage to feed farm animals; data about feed standards for MCs intake by livestock are scarce and insufficient. An increasing risk to farm animals’ health is likely when MCs concentrations in drinking water exceed 4.2 µg·L^−1^ for cattle weighing 800 kg on average and drinking 85 L of water on a daily basis, according to the Australian guideline for fresh and marine water quality [68]. That is to say, the tolerable daily intake (TDI) of MCs for cattle is 0.45 µg per kilogram of body weight per day. Cattle consume, on average, 4% of their live weight in dry matter daily. That is to say, 32 kg of faba bean silage. The EDI of MCs was evaluated by the following equation:

EDI _faba bean_ = MC_32 kg_^−1^/BW_800 kg_, Where MC_32 kg_ represents the amount of MC accumulated per 32 kg of faba bean dry mass and BW_800 kg_ is the body weight of the farm animal (cattle). (6)

### 5.11. Statistical Analysis 

The experimental design was a randomized complete block. Data regarding the growth and photosynthesis parameters related to the SL, TNN, DWS, DWR, Chlorophyll fluorescence and stomatal conductance were means of 10 replicates per treatment and parameters related to the nitrogen, total chlorophyll and MCs contents were means of three replicates per treatment. Data were analyzed by variance analysis (ANOVA) and the mean separation was achieved by LSD test using the COSTAT software. Differences were considered significant at the probability level of *p* ≤ 0.05.

## Figures and Tables

**Table 1:** Effect of microcystins (MCs) crude extract (2.5 mg·L^−1^ of total MCs) for 28 days on growth parameters (SDW: shoots dry weight, RDW: Roots dry weight and SL: shoot length) and nodulation (TNN: total nodules number) of *Vicia faba* plants submitted to three independent exposure experiments under natural environmental conditions. Expressed values are means of 10 replicates. Significant differences (*p* ≤ 0.05) between control and treatment groups are indicated by (*).

Plant Organ	Soils	MC-LR (mg·L^−1^)	Parameters
Shoots			SL (cm organ^−1^)
	Intact	0	20.76 + 1.67
		2.5	20.93 + 1.45
		% Change	−0.84
	Sterilized	0	22.12 + 1.69
		2.5	15.27 + 1.42 *
		% Change	30.96
	Inoculated	0	23.95 + 0.73
		2.5	21.65 + 0.6 *
		% Change	9.6
Shoots			SDW (g organ^−1^)
	Intact	0	0.45 + 0.05
		2.5	0.47 + 0.07
		% Change	−4.11
	Sterilized	0	0.4 + 0.07
		2.5	0.37 + 0.05
		% Change	7.75
	Inoculated	0	0.53 + 0.05
		2.5	0.46 + 0.04 *
		% Change	13.46
Roots			RDW (g organ^−1^)
	Intact	0	0.18 + 0.05
		2.5	0.18 + 0.03
		% Change	0
	Sterilized	0	0.18 + 0.05
		2.5	0.17 + 0.04
		% Change	5.55
	Inoculated	0	0.33 + 0.05
		2.5	0.34 + 0.05
		% Change	−3.03
Nodules			TNN. plant^−1^
	Intact	0	16 + 3.55
		2.5	23.25 + 3.09 *
		% Change	−45.31
	Inoculated	0	50.75 + 11.72
		2.5	50 + 7.39
		% Change	1.47

**Table 2:** The total content of MCs in shoots and roots, bioconcentration factor in shoots (BCF), estimated daily intake (EDI) and the factor exceeding the tolerable daily intake (TDI) (The TDI for humans and farm animals (cattle) is limited in 0.04 and 0.45 µg MC-LR Kg^−1^ of body weight respectively) of faba bean plants treated with 2.5 mg·L^−1^ MCs in different exposure experiments.

	MCs in Water (µg·L^−1^)	MCs in Roots (µg·kg^−1^ DW)	MCs in Shoots (µg·kg^−1^ DW)	BCF 10^−3^	EDI		Factor Exceeding TDI	
					Humans	Farm Animals	Humans	Farm Animals
Intact soil	0	Nd	nd	-	-	-	-	-
	2500	25.1	8	3.2	0.053	0.32	1.32	0.7
Sterilized soil	0	Nd	nd	-	-	-	-	-
	2500	23.8	13.1	5.2	0.087	0.52	2.18	1.16

nd: not detected.
